# *Cryptosporidium* infection in calves and the environment in Asembo, Western Kenya: 2015

**DOI:** 10.11604/pamj.supp.2017.28.1.9313

**Published:** 2017-11-04

**Authors:** Allan Ogendo, Mark Obonyo, Peter Wasswa, Austine Bitek, Amos Mbugua, Samuel Mwangi Thumbi

**Affiliations:** 1 Jomo Kenyatta University of Agriculture and Technology, College of Health Sciences, Kenya; 2 Ministry of Health, Kenya Field Epidemiology and Laboratory Training Program, Kenya; 3 Ministry of Agriculture, Livestock and Fisheries, Directorate of Veterinary Services, Kenya; 4 African Field Epidemiology Network, Kampala, Uganda; 5 Kenya Zoonotic Disease Unit, Kenya; 6 Paul G. Allen School for Global Animal Health, Washington State University, USA

**Keywords:** *Cryptosporidium*, zoonotic, calves, Kenya

## Abstract

**Introduction:**

*Cryptosporidium* species, a zoonotic enteric coccidian parasite, is among the leading causes of diarrhea in children. We evaluated the prevalence of *Cryptosporidium* infections in calves, factors associated with calf infection, environmental contamination of manure by *Cryptosporidium* and factors that expose humans to zoonotic transmission in Asembo.

**Methods:**

in a cross-sectional study conducted from January to July 2015, we collected fecal specimens from 350 randomly selected calves aged ≤ 6 months old and 187 manure samples from the same farms. We assessed farmers’ knowledge about *Cryptosporidium* and collected data on characteristics using structured questionnaires. Modified Ziehl Nielsen staining was used to detect *Cryptosporidium* oocysts from calves’ stool and manure. The prevalence of infected calves and 95% confidence interval (CI) were calculated. Odds ratios (OR) and 95% (CI) were calculated to identify possible factors associated with *Cryptosporidium* infection; multivariable logistic regression performed to identify factors independently associated with the presence of *Cryptosporidium*.

**Results:**

calves’ fecal *Cryptosporidium* prevalence was 8.3% (95% CI: 5.7-11.8) and 7.5% (95% CI: 4.2-12.2) in manure. Odds of infection was higher in calves with loose stool compared to those with normal stool (AOR = 6.1, 95% C.I: 2.2-16.9), calves ≤ 2 months old compared to older calves (AOR=12.7, 95% C.I: 4.5-35.8) and calves in poor sanitation compared to calves in good hygienic conditions (AOR = 9.9, 95% C.I: 3.1-30.7).

**Conclusion:**

presence of *Cryptosporidium* species in calves and environment and reported human contact with animals increases zoonotic risk. We recommend further studies that determine specific *Cryptosporidium* species infecting animals and humans which would better estimate risk of disease transmission to humans.

## Introduction

*Cryptosporidium* species, coccidian parasites of the phylum Apicomplexa, are known to cause diarrhea in humans and animals globally [1]. Over the last 30 years, *Cryptosporidium* species have continued to gain public health importance as the cause of emerging zoonotic infections worldwide. Cryptosporidiosis accounts for up to 20% of all cases of childhood diarrhea in developing countries, and has been associated with an increased risk of death in children aged < 2 years [2,3]. Diarrhea caused by *Cryptosporidium* species infection is typically self-limiting in immunocompetent hosts, but may be severe and life-threatening in immunocompromised individuals such as those with acquired immune-deficiency syndrome (AIDS) or severe malnutrition. There is no effective treatment against cryptosporidiosis and only symptomatic therapy is recommended [4].

Humans and animals become infected by direct ingestion of oocysts, which are shed in the stool of infected animals or other humans. These oocysts can also contaminate water [4]. *Cryptosporidium* can cause outbreaks, as indicated in 1993, when a massive waterborne *Cryptosporidium* outbreak affected more than 400,000 people in Milwaukee, USA [5].

At least 22 species of *Cryptosporidium* have been identified. Of these, *Cryptosporidium* parvum and *Cryptosporidium* hominis are the most important species due to their widespread distribution [6,7]. *Cryptosporidium* parvum has been reported as the main zoonotic species that affects cattle and is the major cause of watery diarrhea in calves worldwide. *Cryptosporidium* hominis is known to be maintained in human-to-human cycles. However, it was detected in calves in Kenya hence considered anthroponotic [8]. Experimental studies on laboratory animal models found that *Cryptosporidium* might lead to growth impairment which would impact production [9].

Various studies in sub-Saharan Africa have reported *Cryptosporidium* prevalence in animals ranging from 2.2% to 35% in cattle [10,11]. Infected cattle and contaminated manures from cattle can be sources of *Cryptosporidium* infection to humans [12]. Some factors related to *Cryptosporidium* infection in these studies and other studies included poor hygiene, calves aged below 3 months, wet seasonality, not separating calves from adult cows, lack of feeding colostrum within the first few hours of life, and intensive production system [11,13,14]. Studies show that younger calves are more vulnerable to *Cryptosporidium* infection [11].

There are few studies of *Cryptosporidium* species infection in cattle in Kenya. However, studies have been undertaken in Kenya that involve children, HIV positive individuals and surface waters [15-18]. In Asembo, Kenya, with an HIV prevalence of 15.4%, one study reported 9% prevalence of *Cryptosporidium* in children [3]. In this location, the burden of cryptosporidiosis in animals has not been evaluated, which may contribute to infections in the human population [19].

The objectives of this study were to estimate the prevalence of *Cryptosporidium* species in calves and their environment, to assess factors associated with infection in calves, and to assess human practices that might predispose humans to zoonotic transmission.

## Methods

We conducted this cross-sectional survey in Asembo area, Rarieda sub-county in Siaya County of western Kenya. Using the Cochran’s (1977) formula for simple random sampling, we estimated that 350 calves would need to be sampled (1 per household) to find a 35% prevalence of calves with *Cryptosporidium* oocysts and assuming a power of 80% and precision of 0.05. The sampling frame, a list of all households that owned calves aged six months and below in each of Asembo’s locations, was obtained from an ongoing population based animal syndromic surveillance study in the area [20]. We used systematic random sampling, for which households with calves aged six months and below were regarded as the primary sampling units. In households with more than one calf meeting the selection criteria, the youngest calf was picked.

Data collection occurred between January and July which has both dry and wet seasons in the study area. During the visit to each selected household, a fecal specimen was collected directly from the selected calf into a sterile, airtight, 10ml plastic tube and transported to the laboratory in a cool box. Presence of *Cryptosporidium* species oocysts in the fecal samples was detected using the modified Ziehl-Neelsen staining technique as described by Clarke and McIntyre [21]. Approximately 50gm of thoroughly mixed manure was collected from 187 randomly selected household among the 350 enrolled households using a sterile plastic tube and transported to the laboratory in a cool box.

A pre-tested structured questionnaire was administered to each household head. The questionnaire collected information about the possible risk factors for *Cryptosporidium* infection for calves, which included age of the calf, consistency of feces (whether normal or loose), level of hygiene, herd size, calf housing, manure handling and uses, sources of water, season (whether wet or dry) and household practices that might lead to human exposure to the pathogen (e.g. contact with animal manure) and general knowledge about the disease. To assess knowledge of cryptosporidiosis, the clinical picture of the diarrhea due to cryptosporidiosis (yellowish, watery and containing mucus) in humans was described to the interviewee in their local language.

Hygiene level was estimated based on the frequency of manure removal, frequency of cleaning of the animals’ sleeping area and presence of slurry on the floor. From these factors, we developed a scoring system with two categories, i.e. good/moderate and poor. Places that were cleaned daily and that appeared dry with very little observable slurry were considered good/moderate hygiene level, whereas those which appeared to be generally wet, dirty and with slurry were categorized as poor.

We also assessed the frequency, with which children, being at higher risk of infection, came into contact with the animals. Contact with animals was assessed by asking the household head whether the children played within the animal sheds or with the animals or whether they participated in any activity e.g. feeding, slaughtering or cleaning the animals. Frequent contact was classified as contact with the animals at least three days in a week.

Data from questionnaires on calf level, herd-level factors and human factors were entered, cleaned and analyzed using Epi-Info™ 7. Frequencies and proportions were calculated for the categorical variables and measures of central tendency and dispersion for the continuous variables. We calculated odds ratio (OR) and 95% confidence interval (CI) for associations between the presence of *Cryptosporidium* oocysts and potential factors. We performed logistic regression to examine independent factors, for which factors with p-value ≤ 0.15 from univariable analysis were included into the multivariable logistic regression model and adjusted odds ratios (AOR) and 95% CIs were calculated. We used a forward step-wise selection method. Factors with p-value ≤ 0.05 were retained in the final model after exploring all statistically and biologically plausible interactions among the variables remaining in the final model.

Protocol approval was obtained from board of post graduate studies of Jomo Kenyatta University of Agriculture and Technology (JKUAT) and ethical clearance referenced as ERC. 1B/VOL.1/167 was obtained from The Jaramogi Oginga Odinga teaching and referral hospital ethics & research committee (JOTRH-ERC) in Kisumu, Kenya. Consent was obtained from owners of the selected households, and each household was given a unique identifier to maintain confidentiality.

## Results

All 350 randomly selected households agreed to participate in the study. We collected fecal specimens from 350 calves aged 6 months and below and 187 environmental manure samples from the identified households. The study calves’ median age was four months (range: 1-6 months). There were 230 samples collected in the dry season (January to March) and 120 samples in the wet season (April to July).

We found a *Cryptosporidium* species prevalence of 8.3% (95% CI: 5.7-11.53) among calves and 7.5% (95% CI: 4.3-11.9) in environmental samples. Most calves, 84.9% (297/350), were from herds with less than 10 animals. The smallest herd had three animals while the largest herd had 19 animals. Among all the sampled calves, 15% (51/350) had visible loose stool at the time of sampling and 33% (116/350) of the calves were reported to have had an episode of diarrhea within the three months preceding the study (Table 1).

**Table 1:** Characteristics of calves assessed for *Cryptosporidium* infection in Asembo, Western Kenya: 2015

Characteristic	Frequency N=350	Percentage (%)
**Age**		
1-3 months	118	34
4-6 months	232	66
**Location**		
East Asembo	86	25
Central Asembo	85	24
West Asembo	91	26
South Asembo	88	25
**Herd size**		
1-10	297	85
11-20≥11	53	15
**Diarrhea status during collection**		
Yes	51	15
No	299	81
**Feces on coat**		
Yes	47	14
No	303	87
**Sampled calf ever had diarrhea in last 3 months**		
Yes	62	18
No	288	83
**Any animal in herd had diarrhea in last 3 months**		
Yes	116	33
No	234	67

There were 274 (78%) households that reported using animal manure as fertilizer on their crops and 75 (22%) used animal manure for building purposes (pasting on walls and floors of mud houses). There were 280 (80%) respondents who reported washing their hands after handling manure and 190 (55%) reported that they removed manure on need basis while only 23 (7%) removed manure from animal sleeping areas daily (Table 2).

**Table 2:** Manure handling and calf management practices in Asembo, Western Kenya: 2015

Factor	Category	Levels	Frequency N=350	Percentage (%)
	Level of hygiene	Good	69	20
		Moderate	187	53
		Poor	94	27
	Mode of calf feeding	Free-grazing	74	21
		Stall-feeding	6	2
		Tethering	269	77
	Calf watering	Calves go to water	96	28
		Water provided at home	254	73
	Source of water for calf	Tap water	41	12
		Rain	59	17
		River/lake	78	22
		Ponds	192	55
	Calf housing	Calf pens	103	29
		Kitchen	112	32
		Open	54	15
		Shed	81	23
	Disinfect calf sleeping area	Yes	41	12
	Access vet services last 3 months	Yes	203	58
	Preventive treatment	Yes	12	3
	Feeding of feed supplements	Yes	102	29
		No	248	71
Manure handling	Heaping	Yes	267	76
	Frequency of manure removal	Daily	23	7
		Weekly	68	19
		Need basis	190	55
		Monthly	43	13
	Uses of manure	Crop	274	78
		Building	76	22
	Hand washing after handling manure	Yes	280	80
	Presence of run-off	Yes	148	42
Season	Season during sample collection	Rainy	120	34
		Dry	230	66

There were 246 respondents (70%) who were not aware of any zoonotic disease that could be acquired by getting into contact with animal feces or manure. Children aged below five years were found in 238 (68%) of the sampled households, out of which 120 (50%) reported frequent contact with animals while 61 (26%) reported no contact with the animals. There were 293 (83%) households that did not restrict animals from accessing water sources that were also used by humans for domestic purposes. In terms of calf housing, 112 (32%) of the calves shared the same housing with humans and did not have calf pens or sheds while 54 (15%) did not have specific sleeping areas and often were tied outside within the compound (Table 2).

At univariable analysis, *Cryptosporidium* infection was associated with presence of loose stool (OR=11.9, 95% CI: 5.3-21.1) compared to normal stool consistency; wet season (OR = 2.2, 95% CI: 1.1-4.7) compared to the dry season and calves aged ≤ 2 months (OR = 12.3, 95% CI: 5.4-28.1) compared to calves > 2 months of age. Calves from households with positive manure samples were at higher risk of being *Cryptosporidium* positive (OR=9.8, 95% CI: 3.1-31.5) compared to calves from households with negative manure samples. In fact, 12 (85%) of the households where manure samples tested positive for presence of *Cryptosporidium* oocysts also had calves that were positive. Calves raised in poor/dirty environments (O.R = 17.2, 95% CI: 6.3-46.8) had higher chances of *Cryptosporidium* infection compared to those raised in good/moderate hygiene conditions. Multivariate analysis identified that calves showing signs of loose stool (AOR = 6.1, 95% CI: 2.2–17.0); reporting poor hygiene at the farm (AOR=10.0, 95% CI 3.3–30.9) and calves being aged two months and less (AOR = 12.9, 95% CI: 4.6–35.8) were significantly associated with *Cryptosporidium* positivity while adjusting for these factors simultaneously (Table 3).

**Table 3:** Crude and adjusted odds ratios for factors related to *cryptosporidium* infection among calves in Asembo, Western Kenya: 2015

Factor	Levels	Cryptosporidium positive n (%)	Cryptosporidium negative n (%)	OR (95% CI)	AOR (95% CI)
Age of calf	≤2 months	17 (59)	33 (10)	12.3 (5.4-28.1)	12.9 (4.6-35.8)
	>2 months	12 (41)	288 (90)	Reference	
Herd size	11-20 calves	8 (28)	45 (14)	2.3 (1.0-5.6)	NS
	1-10 calves	21 (72)	276 (86)	Reference	
Loose stool	Present	17 (59)	34 (11)	11.9 (5.3-27.1)	6.1 (2.2-17.0)
	Absent	12 (41)	287 (89)	Reference	
Presence of other animal reported as diarrheic within herd	Present	19 (66)	97 (30)	4.4 (1.2-9.8)	NS
	Absent	10 (34)	224 (70)	Reference	
Sex of calf	Female	19 (66)	163 (51)	1.8 (0.8-4.1)	
	Male	10 (34)	158 (49)	Reference	
Presence of surface Run-off in the compound	Present	17 (59)	131 (41)	2.1 (0.9-4.4)	NS
	Absent	12 (41)	190 (59)	Reference	
Feeding of calf on commercial supplement	Yes	4 (14)	98 (31)	0.3 (0.1-1.1)	NS
	No	25 (86)	223 (69)	Reference	
Calf watered from Pond	Yes	24 (83)	168 (52)	4.3 (1.6-11.7)	NS
	No	5 (17)	153 (48)	Reference	
Calf Housing type	Other places	15 (52)	223 (69)	0.5 (0.2-1.0)	NS
	Kitchen	14 (48)	98 (31)	Reference	
Hygiene Level	Poor	24	70	17.2 (6.3-46.8)	10.0 (3.3-30.9)
	Good/Moderate	5	251	Reference	

## Discussion

In this present study, we report prevalence of *Cryptosporidium* infection in calves and environment in an area where a previous study had reported high prevalence of *Cryptosporidium* infection among children. Further, we identified factors associated with greater risk of *Cryptosporidium* positivity in calves and their implication in disease transmission and control.

In Asembo, the *Cryptosporidium* prevalence in calves and in environmental manure was similar to a previous study in the same location among children with diarrhea that reported 9% *Cryptosporidium* prevalence in children aged five years and below [3]. Our reported prevalence is within the range for calves reported in another study in Kenya, which reported 7.7% [8]. Poor hygiene level was associated with higher risk of *Cryptosporidium* infection in the calves, which is consistent with other studies [11]. Since *Cryptosporidium* oocysts can survive for long periods in the environment, such unhygienic conditions coupled by the warm and humid climate in Asembo could aid in *Cryptosporidium* persistence and spread in the environment. However, our study found that calves raised under good and moderate hygiene environments were protected from *Cryptosporidium* infection.

This study found that younger calves below two months had greater chances of getting infected with *Cryptosporidium* compared to older calves above two months. This finding was consistent with other studies which also reported similar correlation with age of calves [11,13,22-25]. Young infected calves play an important role in maintaining infection both in the herd and environmental contamination thus representing the greatest zoonotic risk to humans. The prevalence of *Cryptosporidium* in calves in Asembo was lower than the 35% and 78% reported in Tanzania and Canada respectively [11,13]. This observed difference was due to the fact that, in Tanzania only calves aged three months and below sampled were sampled whereas in Canada, only neonatal calves aged 30 days and below and kept under intensive dairy farms were sampled. The age range for the two studies are ages at which calves are at highest risk of infection with *Cryptosporidium*. In Asembo, calves infected with *Cryptosporidium* species were also more likely to have loose stool, similar to other studies carried out in Western France and India [22,23]. However, asymptomatic calves can also shed *Cryptosporidium* oocysts in their feces and because they may be a source of infection, calves should be screened regularly for presence of oocysts [11].

The low awareness on the zoonotic nature of this disease and shared water sources between humans and animals increases chances of water contamination, hence increased risk of zoonotic transmission of *Cryptosporidium* Species to humans. *Cryptosporidium* can easily contaminate water sources as shown in studies on surface waters. High levels of contamination of water sources with *Cryptosporidium* parvum and hominis has been reported in Eastern part of Kenya [18,26]. Water sources for use by humans should therefore be restricted from animal access in order to reduce chances of contamination.

Children aged below 5 years are more vulnerable to *Cryptosporidium* infection according to other studies conducted in Africa [3,27,28]. Their contact with cattle, both infected and uninfected, should therefore be reduced so as to decrease the risk of zoonotic transmission. The practice of housing calves in the kitchens increases closer contact between calves and humans thereby increasing the risk of cross-infection by *Cryptosporidium* species.

Our study showed presence of *Cryptosporidium* oocysts in manure. Most of the people interviewed collected manure into heaps and used it for building and as fertilizer on their food crops. Farmyard manure may contain high numbers of *Cryptosporidium* oocysts and, consequently, water may be contaminated by manure or slurry washed into rivers and vegetable crops may also be contaminated by direct manuring of the fields in which they are grown. Other studies have also shown that contaminated manures from dairy or beef cattle operations can be major sources of *Cryptosporidium* oocysts unless manure management or treatment strategies are used to minimize oocyst viability or transport to water [12]. This poses a serious health hazard as the crops may easily become contaminated with the *Cryptosporidium* oocysts which survive for many years in the environment thereby increasing risk of transmission to humans. Since there was similar prevalence in the calves and the environment and it is easier to sample the latter, environmental sampling may be just as effective as animal sampling in future studies. This is due to the fact that it is more cost effective and causes no stress to the animals. The prevalence reported in such studies can be used as proxy indicators of the prevalence in animals.

The potential of human infectivity by the *Cryptosporidium* oocysts identified in our study could not be established since we used microscopy (mZN) as our tool of diagnosis. The mZN is a widely used screening test for *Cryptosporidium*; however mZN is not specific enough to discern species as would a molecular sequencing test. Not all *Cryptosporidium* species are zoonotic; therefore we could not establish the proportion of zoonotic species among our positive cases, which would have enabled us to quantify the zoonotic risk posed by the infected calves. In addition, the use of mZN might have under-estimated the prevalence of *Cryptosporidium* infection because mZN has lower sensitivity compared to PCR [29]. Future studies should include molecular diagnosis to identify the parasites to species level and correctly quantify the zoonotic potential of this parasite.

## Conclusion

*Cryptosporidium* was present in both calves and environmental manure in Asembo. Our study found that the prevalence was higher among calves raised in poor hygiene levels and in calves with loose stool; however we could not establish whether the loose stool was due to *Cryptosporidium* infection. Infected animals are potential reservoirs for zoonotic *Cryptosporidium* which can infect humans living in close contact with them. Improper handling of animal manure poses a risk of infection to both humans and animals. Since there was an almost similar prevalence of presence of *Cryptosporidium* oocysts in both the calves and the sampled environmental manure, we recommend that when resources are not sufficient to collect rectal feces from calves then environmental sampling can be used to estimate the prevalence. Intervention strategies targeted towards young calves might reduce transmission within herds and into the environment, thereby curtailing the zoonotic pathway. Since there are no effective medications against *Cryptosporidium* infections, maintenance of high hygiene standards remains the surest way of controlling its spread.

### What is known about this topic

- *Cryptosporidium* causes diarrhea in calves, young children and immunocompromised individuals.

### What this study adds

- Assessed human knowledge and practices that might point to the zoonotic risk of transmission posed to humans;
- *Cryptosporidium* species prevalence of 8.3% among calves and 7.5% in environmental samples;
- We found that 293 (83%) households shared same water sources with their animals.

## Competing interests

The authors declare no competing interest.
